# Biosensor Based on the Immobilization of Laccase on β‐Cyclodextrin Membrane for the Evaluation of Antioxidant Capacity in Real Samples

**DOI:** 10.1002/open.202400228

**Published:** 2024-10-21

**Authors:** Jorge Juárez‐Gómez, Omar Alejandro Báez‐Melga, Dafne Sarahia Guzmán‐Hernández

**Affiliations:** ^1^ Departamento de Química Universidad Autónoma Metropolitana Iztapalapa Av. San Rafael Atlixco #186, Col. Vicentina México, D.F. C.P. 09340; ^2^ Conahcyt-Universidad Autónoma Metropolitana Iztapalapa Departamento de Química Av. San Rafael Atlixco #186, Col. Vicentina México, D.F. C.P. 09340

**Keywords:** Biosensor, Antioxidants, β-CD, Electropolymerization, Antioxidant capacity

## Abstract

The optimization of a new amperometric biosensor for evaluating antioxidant capacity in real samples is reported. The biosensor is based on the immobilization of Laccase from *Trametes versicolor* on an electropolymerized β‐cyclodextrin polymeric membrane on a glassy carbon electrode. The process of electropolymerization, which was successful even in the presence of the enzyme, was a key step in biosensor synthesis. Variables such as pH, temperature, and enzyme concentration were optimized using a factorial design with two levels for each factor. Different electrodes were constructed and tested using caffeic acid as a standard. The best biosensor is synthesized at pH 3.0 with 6 mg/mL of enzyme and 30 °C. The biosensor presented a response time of ≤30 seconds and good stability in its amperometric response. The biosensor was used to evaluate the antioxidant capacity of real samples. Infusions of green, black, red, and white tea were assessed. The biosensor showed excellent stability and good performance regarding response time, stability, and easy fabrication. The proposed biosensor is a good option for evaluating antioxidant capacity in real samples without sample pretreatment. It combines a simple fabrication methodology and a minimal extraction process for rapid and reliable phenolic content determination in real samples.

## Graphical abstract

The design and optimization of a new biosensor for the evaluation of antioxidant capacity are presented. The biosensor shows an excellent response to antioxidant standards. The response of the biosensor is reproducible, simple, and inexpensive, showing excellent stability and good response time performance. The new biosensor can be used for the evaluation of antioxidant capacity in real samples without pretreatment.

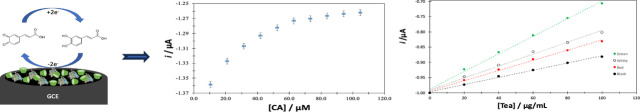

## Introduction

Antioxidant compounds are chemical species that protect biological systems from free radicals that oxidize them, preventing the so‐called oxidative stress.[^1^, ^2^, ^3^] Antioxidants can be classified as primary or secondary depending on their mechanism of action. First‐class antioxidants act as hydrogen donors or free radical acceptors, generating more stable radicals and generally having phenolic structures. Second‐class antioxidants do not generate free radicals in their mechanism of action.^[4]^ Primary antioxidants can be found in nature in fruits, vegetables, and plants.[^5^, ^6^] Therefore, they are the species to which we have the most significant access and the most studied species.

The antioxidant capacity of a species depends on several factors, such as its chemical structure, temperature, and concentration. The structure plays an essential role since the chemical reactivity of antioxidants against free radicals and other reactive oxygen species (ROS) depends on it.^[7]^ There is an excellent variety of ROS, but the most important are hydroxyl radicals (HO^−^), superoxide radicals (O_2_^−^), radical nitrogen dioxide (NO_2_^−^), and radical nitrogen oxide (NO^−^) because they are the species that are formed in the most significant quantity both in the environment and cells.[^5^, ^7^, ^8^]

Antioxidant capacity is a physicochemical parameter that can be evaluated for a specific antioxidant species. However, when talking about real samples, what is evaluated is an equivalent antioxidant capacity (EAC) using a particular antioxidant species as a standard; for example, when trolox is used as a standard, the equivalent antioxidant capacity to Trolox (TEAC) is evaluated, and if caffeic acid is used as a standard, the equivalent antioxidant capacity to caffeic acid (CAEAC) is evaluated. Clearly, it is difficult to isolate all the antioxidant species present in a real sample and to determine the specific antioxidant capacity of each one, which leads to the conclusion that in EAC, there is a strong synergy between the antioxidant species.

Methods for evaluating antioxidant capacity are diverse in mechanism and instrumentation.[^9^, ^10^] There are spectrophotometric, fluorometric, electrochemical, and chromatographic methods for estimating antioxidant activity in real samples. However, the most widely used are spectrophotometric and electrochemical methods.[^11^, ^12^, ^13^] Among the former is the DPPH method, where antioxidant species react with a relatively stable organic radical (DPPH⋅) and the absorbance of this radical is measured in the visible region of the electromagnetic spectrum;[^14^, ^15^, ^16^] the ORAC method based on the reaction of antioxidant molecules with the peroxyl radical;[^17^, ^18^] the CUPRAC method based on the reduction of Cu(II) to Cu(I) by the antioxidant species.[^19^, ^20^] Among the electrochemical methods, voltammetric methods stand out, where the oxidation current of antioxidant species is measured and related to the antioxidant capacity; it is even possible to generate free radicals in vivo and measure the oxidation current of antioxidants in the presence of these radicals.^[21]^ Another widely used electrochemical method is the amperometric method and, more specifically, the amperometric sensors based on oxidoreductase enzymes immobilized on the surface of a working electrode;^[22]^ its operation is simple: the enzyme oxidizes the antioxidant species, and using a potential imposed on the electrode the oxidized species is reduced recovering its original structure generating in this cycle an electric current. A biosensor for phenolic compounds based on a chemically modified Laccase from *Coriolus hirsuta* immobilized on functionalized screen‐printed carbon electrodes has also been reported.^[23]^ Also, a β‐cyclodextrin‐capped gold nanoparticle surface designed for tyrosinase‐based nanosensors for catechol determination has been proposed.^[24]^

In short, the methods proposed in the literature for estimating the antioxidant capacity in real samples are many and very diverse. However, the scientific community continues to suggest better methods that can help reduce the costs and time needed for analysis. In addition, more methodologies and instruments are generated daily to facilitate the study of real matrices. In most of the reported research, antioxidant capacity is assessed in tea infusions, herbal infusions, or even fruit juices, thus ensuring that the methodology is sufficiently robust. The importance of rapid and reliable methods for evaluating antioxidant capacity is that the main source of antioxidants is obtained from the diet; therefore, the more rapid a methodology is, the more recommendations can be made on the best sources of antioxidants.

In this work, a new amperometric sensor based on the immobilization of Laccase from *Trametes versicolor* (LTv) by entrapment in an electropolymerized β‐cyclodextrin (β‐CD) network is proposed for evaluating CAEAC in infusions of green, white, black, and red tea.

## Experimental Section

### Materials and Methods

All reagents were analytical grade and supplied by Sigma–Aldrich. 2‐hydroxypropyl‐β‐cyclodextrin (≥97 %). The Laccase from *Trametes versicolor*, LTv (EC 420‐150‐4) 0.87 IU/mg. KH_2_PO_4_ and K_2_HPO_4_ were used to prepare 0.05 M phosphate buffer at pH 3.0 and 5.0. Also, 0.1 M acetate buffer at pH 4.5 was prepared with sodium acetate and acetic acid. Caffeic acid (CA), pyrocatechol (PC), and gallic acid (GA) stock solutions were prepared in methanol. Subsequently, 0.1 M, 0.01 M, and 0.001 mM stock solutions were freshly prepared by suitable dilutions the day they were used and kept in a dark flask at 4 °C, half immersing them on an ice tray during measurements.

### Electropolymerization

**Figure 1:**
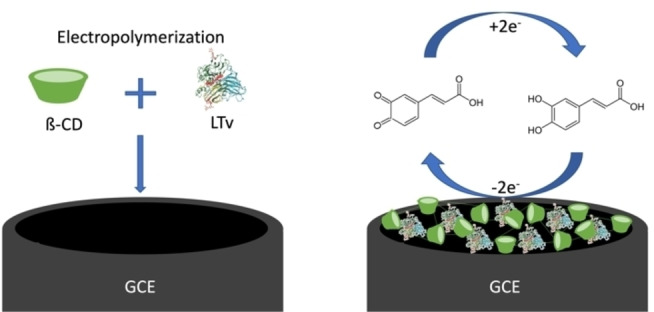
Graphical representation of LTv enzyme immobilization on electropolymerized β‐CD membrane and biosensor performance.

Different solutions containing 2‐hydroxypropyl‐β‐cyclodextrin (β‐CD) 6 mM and LTv 4 mg/mL in 0.05 M phosphate buffer pH 5.0 were prepared. The solution was placed in a three‐electrode thermostatted electrochemical cell. A glassy carbon electrode (GCE) was used as a working electrode, Ag/AgCl (3 M) was used as a reference, and a platinum wire was used as an auxiliary electrode. Before synthesizing the sensing membrane, the working electrode was polished in 0.3 μm alumina for 20 min and washed with deionized water and ultrasonic. The electrode was then electrochemically cleaned in 0.5 M sulfuric acid by cyclic voltammetry from −1.0–2.0 V at 0.1 V/s for 20 cycles using a BAS‐Epsilon potentiostat. For the electropolymerization of β‐CD, the cyclic voltammetry technique was used in a potential window from −2.0–2.2 V at a sweep rate of 0.1 V/s for 30 cycles with constant stirring. A representation of the enzyme immobilization on the polymeric membrane is shown in Figure 1.

### Amperometric Measures

The amperometric response of the constructed sensors was measured using a BAS‐Epsilon potentiostat using the chronoamperometry technique, imposing a reduction potential of −0.2 V. All amperometric experiments were performed in a thermostatted electrochemical cell using the synthesized biosensors as the working electrode, Ag/AgCl (3 M) as the reference, and platinum as the auxiliary electrode. For each experiment, 10 mL of 0.1 M acetate buffer, pH 4.5, was added, and the thermostat was adjusted to the desired temperature. Finally, successive aliquots of CA, GA, or PC standard solutions were added.

### Spectrophotometric Measurements

The activity of the free enzyme was determined spectrophotometrically in a PerkinElmer Lambda 20 apparatus. The absorbance of the reaction product between LTv (8 mg/mL in 0.1 M acetate buffer and pH 4.5) and different concentrations of CA was measured. The initial rate of the enzymatic reaction was determined by the change in absorbance with respect to time for each substrate concentration.

### Membrane Optimization

The sensing membrane was optimized using a 2^3^‐factorial design (two levels, three factors). The effect of pH (3.0 and 5.0), enzyme concentration (4 and 6 mg/mL), and temperature (20 and 30 °C) on the biosensor response was measured by analyzing the sensitivity, detection limit, linear range, and dispersion data.

### Analysis in Herbal Infusions

Tea (*Camellia sinensis*) samples were obtained from EUROTE. Infusions of green, white, black, and red tea were prepared by placing 1.0 g of each tea in 100 mL of water at 98 °C for 4 minutes. The infusion was filtered, and upon reaching room temperature, successive additions of each were made over an electrochemical cell thermostatized at 30 °C containing 10 mL of 0.1 M acetate buffer pH 4.5. The amperometric measurements were performed with the electrode that presented the best performance parameters, using Ag/AgCl as a reference and Pt as an auxiliary electrode.

## Results and Discussion

### Enzyme Immobilization

**Figure 2:**
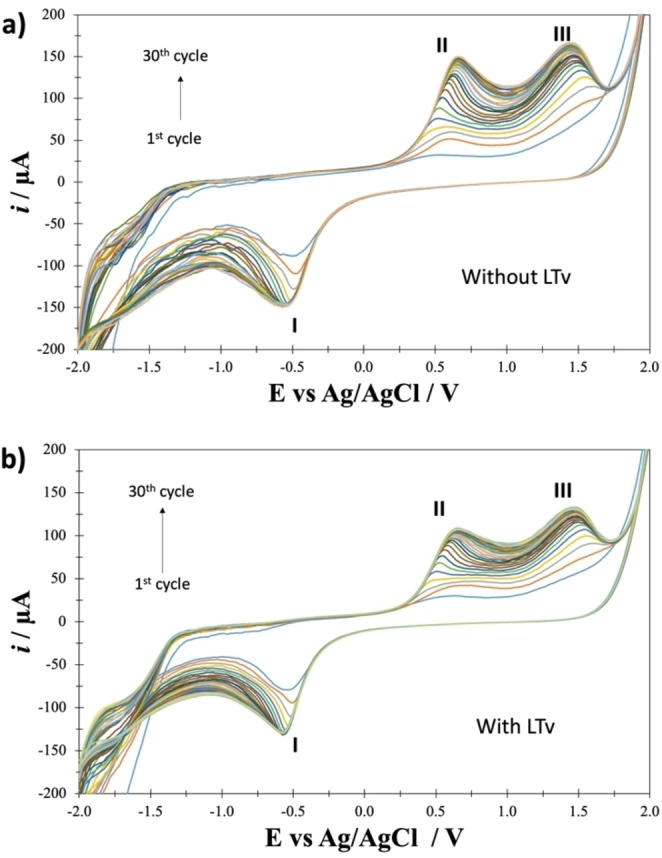
a) Cyclic voltammograms for the system 6 mM β‐CD; b) Cyclic voltammograms for the system 6 mM β‐CD and 4 mg/mL LTv, on a GCE in 0.05 M phosphate buffer pH 5.0 media with a successive scan program in the range of −2.0–2.0 V at 100 mV/s scan rate in anodic direction.

LTv was immobilized by entrapment on a β‐CD polymeric membrane. The working electrode modification was carried out by cleaning with a solution of 0.5 M H_2_SO_4_ within the −1.0 V–2.0 V potential window at 100 mV/s potential scan rate, applying 20 cycles, and then successive cyclic voltamperometry was performed with a solution of β‐CD 6 mM and LTv 4 mg/mL at pH 5.0, applying 30 cycles within the −2.0 V–2.2 V potential window at 100 mV/s potential scan rate. Therefore, the electrode thus modified was termed the GCE‐βCD‐LTv biosensor (see Figure 2).

It is possible to observe that, from the first cycle, there is a cathodic peak (I) at approximately −0.5 V. For the second cycle, two anodic peaks are formed at approximately 0.6 V (II) and 1.5 V (III). The peak current increases with each cycle, indicating that the β‐CD polymeric membrane is forming on the glassy carbon surface. It is also observed that the current increases rapidly until the ninth cycle, and by the tenth cycle, the increase is already much more minor. Pereira et al.^[25]^ point out that the cathodic peak (I) is directly related to the success of the polymer synthesis, i. e., the higher the definition and intensity obtained in the peak (I), the more electropolymerization is guaranteed. Figure 2a shows the family of cyclic voltammograms for the electropolymerization of β‐CD in the absence of enzymes. Figure 2b shows the same family of cyclic voltammograms, but these results show that electropolymerization is carried out even in the presence of the LTv enzyme. It is possible to observe that peaks I, II, and III occur at practically the same potentials in the presence of the enzyme, in addition to the fact that the potential drop, when β‐CD is cycled in the presence of the enzyme, occurs at potentials lower than −1.5 V, while in the absence of the enzyme, the electropolymerization must be brought to a potential of −2.0 V.

### Interaction Study of Caffeic Acid (CA) and Pyrocatechol (PC) on GCE and GCE‐βCD‐LTv

**Figure 3:**
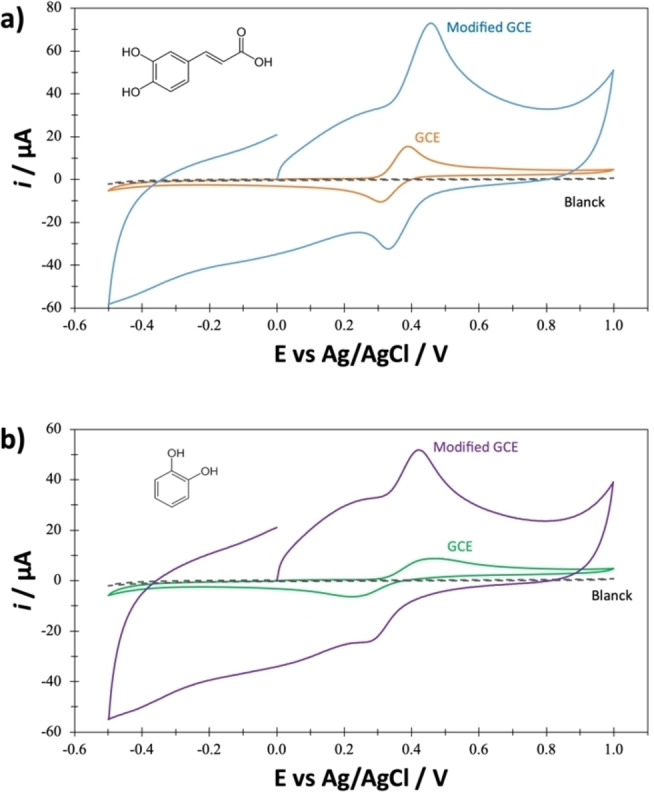
Experimental cyclic voltammetry recorded at 100 mV/s in the GCE/0.1 M acetate buffer system (pH 4.5) for CA (a) and PC (b) on an electrode modified an unmodified.

Figure 3 shows the cyclic voltammograms for CA (3a) and PC (3b) on a GCE vs. Ag/AgCl in a solution acetate buffer 0.1 M pH 4.5 at 1.0×10^−4^ M concentrations of CA and PC, respectively, within the 0.5 V–1.0 V potential window at a 100 mV/s potential scan rate. It is possible to note in Figure 3a the presence of an anodic peak at approximately 0.4 V, corresponding to the oxidation of CA, and a cathodic peak at 0.25 V, corresponding to the reduction of the oxidized CA. In comparison, PC presents an anodic peak at 0.5 V and a cathodic peak at 0.22 V, corresponding to the reduction of the oxidized PC (see Figure 3b). Cyclic voltammograms can also be observed under the same conditions using the GCE‐βCD‐LTv biosensor. In this case, the oxidation‐reduction potentials of the standards do not show a significant difference. However, the anodic and cathodic peak currents substantially grow when the electrode is modified. The experimental results demonstrate that the β‐CD membrane increases the electrode's sensitivity and improves the biosensor's performance parameters. Therefore, a reduction potential imposed at −0.20 V will reduce the species oxidized by the enzyme in real samples.

### Amperometric Response of the GCE‐βCD‐LTv Biosensor

**Figure 4:**
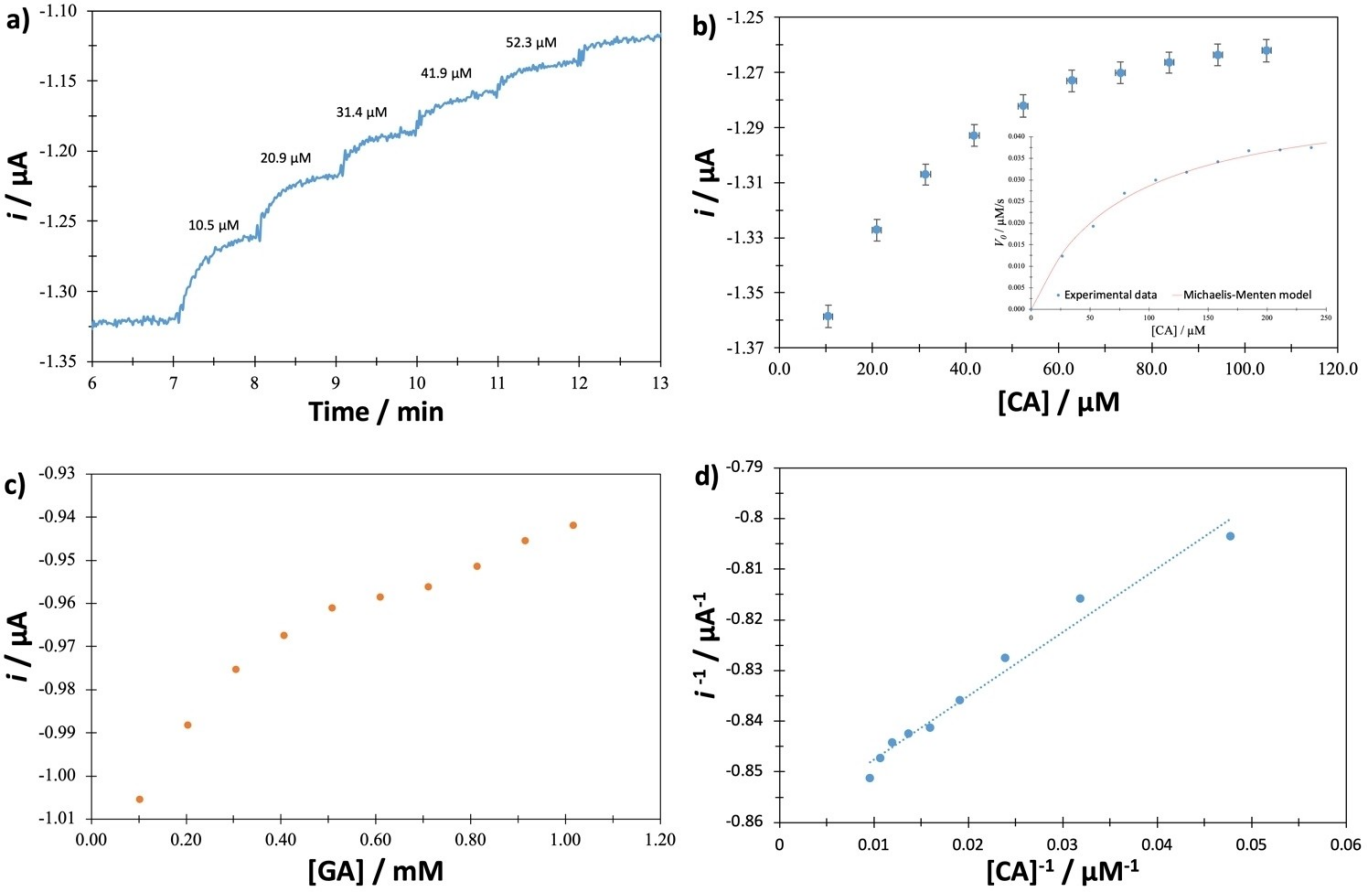
a) Chronoamperometric response of the GCE‐βCD‐LTv biosensor at an imposed potential of −0.2 V, b) Graph *i*=*f*([CA]) obtained from biosensor data, c) Graph *i*=*f*([GA]) and d) Graph *i* ^*−1*^=*f*([CA]^*−1*^) obtained from GCE‐βCD‐LTv biosensor. All experiments were performed in 0.1 M acetate buffer pH 4.5 and 20 °C.

The amperometric response of the GCE‐βCD‐LTv biosensor was tested against standards such as CA and GA. The response to these studies is presented in Figure 4. Figure 4a shows the amperometric response as a function of time for different concentrations of CA. It can be observed that the biosensor presents a stable response over time, but when CA is added, the current increases until it stabilizes in approximately one minute; this behavior is similar for each addition of the standard. Figure 4b shows a graph of the current as a function of CA concentration, and behavior is observed that follows the Michaelis‐Menten kinetic model; this agrees perfectly with that obtained by spectrophotometry when the enzyme is free (*V_max_=0.051* μM/s, *K_m_=*76.28 μM). When CA is used as substrate, the enzyme starts to saturate around 60 μM concentration; however, when GA is used as substrate (Figure 4c), the enzyme does not clearly show saturation. Figure 4d shows the Lineweaver‐Burk plot of double reciprocal, and the data present a linear trend; this demonstrates that the kinetics presented by LTv immobilized in the polymeric network of β‐CD can be represented by the Michaelis‐Menten kinetic model. The kinetic parameters obtained for the immobilized enzyme are *V_max_=1.23* μM/s and *K’_m_=*41.61 μM. The apparent Michaelis constant is lower than when the enzyme is free, thus demonstrating that the affinity of LTv increases when immobilized on the β‐CD polymeric membrane. The increase in the maximum velocity and the decrease in the Michaelis constant suggest that the substrate can reach the enzyme's active site more easily.

### Sensing Membrane Optimization

The sensing membrane was optimized using a 2^3^‐factorial design (two levels, three factors). The factors studied were pH, enzyme concentration, and temperature in the electropolymerization. The factors were chosen because enzyme activity is susceptible to changes in pH and temperature. However, concentration also plays an important role since the number of active sites available for catalysis to take place depends on it. Table 1 shows the levels used for each factor in the optimization.

**Table 1:** Levels imposed for each factor in the experimental design for the sensing membrane optimization.

Factor	Experimental values	
	Level (−)	Level (+)
X_1_: pH	3	5
X_2_: LTv (mg/mL)	4	6
X_3_: Temperature (°C )	20	30

Table 2 shows the eight experiments proposed in the experimental design for sensing membrane optimization. The pH of each experiment was buffered with acetate buffer solutions. In this experimental design, eight biosensors were constructed with the factors and levels described above. The amperometric response of each biosensor was evaluated using CA as a standard; all amperometry was carried out in 0.1 M acetate buffer, pH 4.5, and 30 °C.

**Table 2:** Proposed experiments for the sensing membrane optimization.

Experiment	Level matrix			Experiment matrix		
	1	2	3	X_1_	X_2_	X_3_
1	–	–	–	3	4	20
2	+	–	–	5	4	20
3	–	+	–	3	6	20
4	+	+	–	5	6	20
5	–	–	+	3	4	30
6	+	‐	+	5	4	30
7	–	+	+	3	6	30
8	+	+	+	5	6	30

All electrodes were evaluated in triplicate, and the average result is shown in Table 3. The lowest sensitivity was obtained by biosensor 4 with (0.730±0.051) μA/mM, and the highest sensitivity was obtained by biosensor 7 with (2.38±0.15) μA/mM. The best limit of detection (LOD) and the best limit of quantification (LOQ) were shown by sensor 7. Therefore, the GCE‐βCD‐LTv‐7 biosensor was selected for testing in real samples. It should be clarified that the sensing membranes were synthesized at pH 3.0 and 5.0 according to the factorial design (see Table 1) using phosphate buffer. However, amperometric tests of each biosensor constructed were performed in an acetate buffer. The catalytic parameters of LTv are maximized at 30 °C, and it has been demonstrated when the pH is buffered to 4.5 with acetates.^[22]^

**Table 3:** Analytical parameters of each biosensor constructed (n=3). Experimental conditions for all sensors were 0.1 M acetate buffer, pH 4.5, and 30 °C.

Biosensor	Slope/μA/mM	LOD/μM	LOQ/μM	Linear range/μM	R^2^	s_y_	CV/%
GCE‐βCD‐LTv‐1	1.35±0.13	9.37	31.22	9.37–62.81	0.9740	0.00421	17.8
GCE‐βCD‐LTv‐2	1.59±0.20	7.89	26.29	7.89–35.65	0.9426	0.00417	1.0
GCE‐βCD‐LTv‐3	1.96±0.18	7.52	25.07	7.52–40.85	0.9587	0.00491	4.4
GCE‐βCD‐LTv‐4	0.730±0.051	5.69	18.95	5.69–35.75	0.9760	0.00138	18.1
GCE‐βCD‐LTv‐5	1.38±0.34	11.99	39.96	11.99–30.81	0.8462	0.00553	2.3
GCE‐βCD‐LTv‐6	2.05±0.21	6.50	21.66	6.50–41.07	0.9609	0.00445	5.1
**GCE‐βCD‐LTv‐7**	**2.38±0.15**	**4.06**	**13.53**	**4.06–41.74**	**0.9849**	**0.00322**	**0.9**
GCE‐βCD‐LTv‐8	2.05±0.14	4.40	14.66	4.40–41.74	0.9823	0.00300	8.8

**Figure 5:**
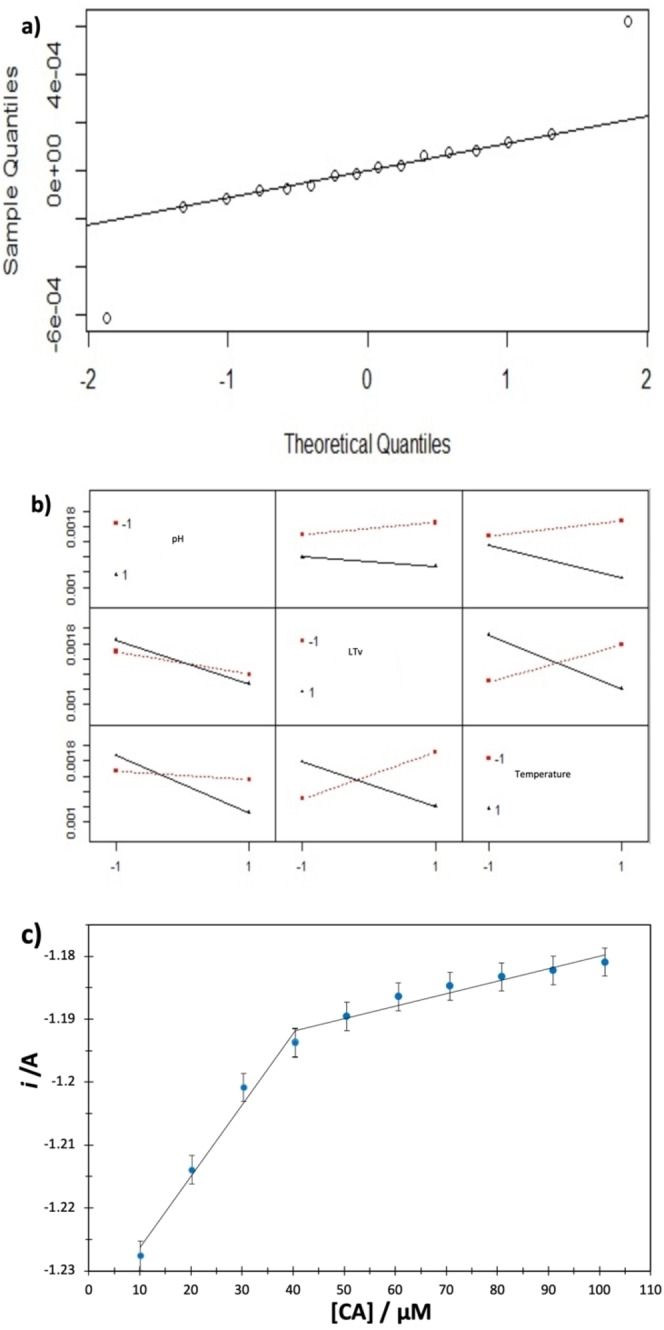
Quartiles (a) and interaction (b) plot for the experiments of the applied factorial design, c) Graph *i*=*f* ([GA]) obtained from biosensor GCE‐βCD‐LTv‐7 data.

Figure 5 shows the analysis of the factorial design variables and the amperometric response of biosensor GCE‐βCD‐LTv‐7. Figure 5a shows the quartiles plot for the experiments of the applied factorial design, and a linear trend is observed, indicating that the experimental data follow a normal distribution. pH was the most critical factor in biosensor synthesis, followed by temperature, and there appears to be no significant difference when the enzyme concentration varies. The interaction study, see Figure 5b, shows an interaction between pH and enzyme concentration and between pH and temperature. However, it shows a strong interaction between temperature and enzyme concentration. Therefore, controlling variables such as pH and temperature are crucial for the biosensor to function adequately. Figure 5c shows the amperometric response for biosensor seven against different CA concentrations. Biosensor 7 presents two zones where the current increases linearly with CA concentration with less than 5 % coefficients of variation.

### Antioxidant Capacity in Real Samples

**Figure 6:**
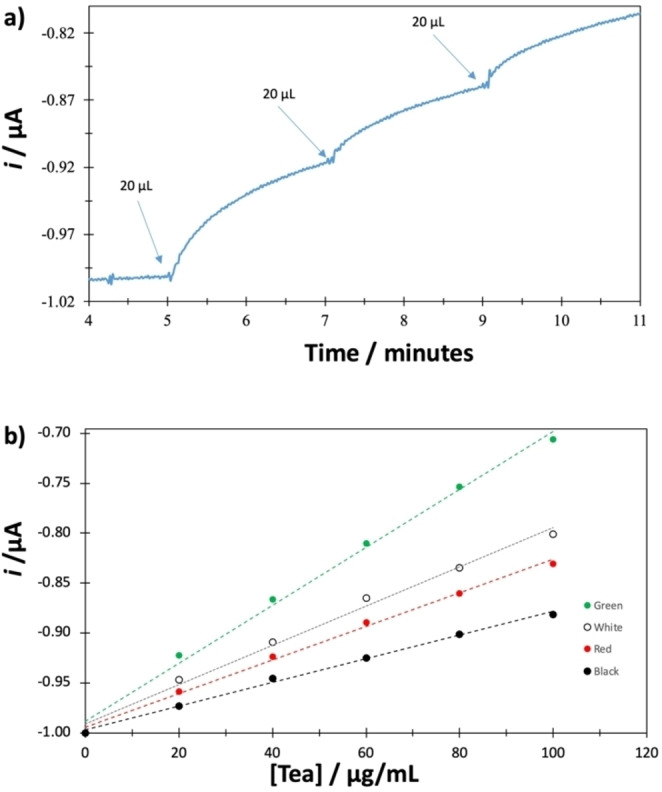
a) Amperometric response of the GCE‐βCD‐LTv‐7 biosensor against green tea additions. b) Calibration curves of the biosensor for different real samples.

The GCE‐βCD‐LTv‐7 biosensor was used to evaluate the antioxidant capacity of different real samples. Infusions of green, red, black, and white tea were prepared by placing 1.0 g of leaves of each tea in 100 mL of water at 98 °C for 4 minutes. Each infusion was filtered, and upon reaching room temperature, additions were made in an electrochemical cell containing 10 mL of 0.1 M acetate buffer (pH 4.5) at 30 °C. The GCE‐βCD‐LTv‐7 biosensor was used as a working electrode, Ag/AgCl as a reference, and platinum as an auxiliary electrode. Figure 6a shows the biosensor's amperometric response against different green tea infusion additions. It is observed that the reduction current remains constant until the addition of the first 20 μL of infusion, then the current increases until it stabilizes. Figure 6b shows the calibration curves of the biosensor for the different real samples. The biosensor shows a linear response of the reduction current as a function of tea concentration. Therefore, these results can be used to estimate the antioxidant capacity of infusions of real samples. Green tea presented a higher response, followed by white, red, and black tea. The amperometric results suggest that green tea has higher antioxidant activity.

The caffeic acid equivalent antioxidant capacity (CAEAC) was evaluated by relating each tea's response to the standard's response. Table 4 shows the results of the CAEAC expressed in millimoles of caffeic acid per milligram of tea. The antioxidant capacity calculated for each tea sample confirms that green tea has the highest antioxidant activity, followed by white, red, and black tea.

**Table 4:** Antioxidant capacity was evaluated for different tea infusions. The results are measured in mmol of caffeic acid per mg of tea.

Tea	Slope/μA*mL/μg	CAEAC/mmol/mg
Green	0.002900	1.415
White	0.001963	0.957
Red	0.001681	0.820
Black	0.001184	0.578

This analysis suggests that green tea leaves contain more phenolic compounds than other teas. The treatment carried out to obtain the different types of tea is a parameter that has an essential influence on the antioxidant content. The extraction of phenolic compounds may be easier from green tea leaves. These results agree favorably with research reporting the content of phenolic compounds in tea extracts.^[26]^ Therefore, the proposed biosensor shows reliable results in evaluating the antioxidant capacity of infusions of real samples.

## Conclusions

The immobilization by entrapment of LTv on a β‐CD polymeric membrane on the surface of a glassy carbon electrode was successfully carried out. With the immobilization of LTv on the polymeric membrane, a biosensor for evaluating antioxidant capacity in standards and real samples was optimized. The GCE‐βCD‐LTv biosensor constructed by enzymatic immobilization exhibited an excellent response for quantifying polyphenols. The proposed immobilization process provided a matrix capable of retaining the enzyme and allowing the diffusion of the antioxidant species through the polymeric film. Experimental conditions have an essential influence on the performance and sensitivity of the biosensor. The pH was the most important variable to control in the experimental design. The biosensor showed excellent stability and good performance regarding response time, stability, and easy fabrication. The proposed biosensor is a good option for evaluating antioxidant capacity in real samples without sample pretreatment. Finally, the proposed biosensor presented remarkable advantages over other biosensors. Its low cost, ease of construction, ease of handling, ease of operation, ease of storage and transportation, good performance, and short response time make this electrode a good option for easy, fast, and reliable phenolic content determination in real samples.

## Author Contributions

Juárez‐Gómez: Writing – original draft, Visualization, Methodology, Funding acquisition, Data curation, Conceptualization, review, editing, Supervision & Conceptualization. Báez‐Melga: Writing – original draft, Investigation. Guzmán‐Hernández: Visualization, Review & editing.

## Conflict of Interests

The authors declare that they have no known competing financial interests or personal relationships that could have appeared to influence the work reported in this paper.

1

## Data Availability

The data that support the findings of this study are available in the supplementary material of this article.
